# Determination of tylosin concentration in sow’s milk after intramuscular administration

**DOI:** 10.2478/jvetres-2025-0008

**Published:** 2025-09-30

**Authors:** Piotr Cybulski, Anna Gajda, Aleksandra Kuśmierz

**Affiliations:** 1 Goodvalley Agro S.A., 77-320 Przechlewo, Poland; 2 Department of Pharmacology and Toxicology, National Veterinary Research Institute, 24-100 Puławy, Poland

**Keywords:** tylosin, UHPLC-MS/MS, milk, sows, pigs

## Abstract

**Introduction:**

Tylosin’s pharmacokinetics has been defined in different species of animals. Despite the description to date of multiple analytical protocols for the determination of tylosin in a variety of biological matrices, research evaluating the antibiotic concentration in sow’s milk cannot be found in scientific literature. This study aimed to conduct such an evaluation.

**Material and Methods:**

The study was carried out on five lactating sows reared on a farm located in Poland. The animals were given intramuscular injections of tylosin at 10 mg per kg of body weight for three consecutive days. Milk samples were collected 3 h and 1, 2, 5, 7, 14 and 21 d after the first administration. The determination of tylosin was carried out using ultra-high performance liquid chromatography with tandem mass spectrometry.

**Results:**

The highest mean concentration of the substance (1,802 μg/L) was found in the samples collected 3 h after the injection. The mean levels of tylosin in the samples collected on day 1 and day 2 were 744 μg/L and 482 μg/L, respectively. The mean concentration on day 5 was lower, falling to 97 μg/L. The value of 6 μg/L in the samples collected on day 7 was the lowest noted. The samples obtained 14 and 21 d after the first administration were below the limit of quantification.

**Conclusion:**

Tylosin easily passes the blood–milk barrier in sows, reaching its maximum concentration in a short time. To the best of our knowledge, this is the first report describing the excretion of tylosin into sow milk.

## Introduction

Initially isolated in 1961, tylosin (Chemical Abstracts System number 1401-69-0) is a traditional representative of the class of macrolides, a significant group of antibiotics secreted by soil-borne bacteria belonging to the *Streptomyces* genus (29). Structurally, the antibiotic consists of a typical macrolactone ring containing 16 carbon atoms (a tylonolide), with mono- and disaccharide branches attached (18). Its trade name has traditionally been used for a mixture of four pharmacologically active derivatives produced by *S. fradiae* through fermentation: tylosin A, the major component of the mixture and most commonly used marker residue; and three minor factors – tylosin B (desmycosin), tylosin C (macrocin), and tylosin D (relomycin) (15, 21). The relative bioactivities of the constituents are 1, 0.83, 0.75 and 0.35, respectively (34).

Similarly to the majority of members belonging to the macrolide class, the antimicrobial mode of action of tylosin is exerted by its reversible binding to the 23S rRNA component of the 50S ribosomal subunit, thus effectively preventing protein synthesis in susceptible bacteria (3). An alkaline environment was found to increase its antimicrobial activity. Tylosin’s pharmacokinetics has been defined in different species of animals (16, 17, 25) and characterised in the available literature by a high volume of distribution throughout tissues and body fluids, after both oral and parenteral administration of the drug. Tylosin binds to plasma proteins at a slight to moderate level. Its half-life in most animal species was defined as 3 to 4 h, and its excretion is mostly *via* faeces and bile (29).

Tylosin presents antimicrobial properties typical of the class. Active against mycoplasma, most Gram-positive bacteria (such as *Streptococcus* and *Staphylococcus* species), and several Gram-negative bacteria, it is described as a medium-spectrum antibiotic. Tylosin is predominantly considered a bacteriostatic antimicrobial agent; however, it was found to have bactericidal properties at elevated concentrations. Therapeutic usage of tylosin is strictly limited to veterinary medicine. Commercially important, the antibiotic has been authorised for marketing in several countries in the forms of tylosin base and its phosphate and tartrate salts, administered *via* intramuscular injection or orally, in the drinking water or after incorporation into medicated feed (29).

Early studies performed on several in-feed antibiotics including tylosin given to commercially reared swine reported significant improvements in both feed efficiency and growth performance of the animals (5). Nevertheless, owing to the well-documented environmental pollution and substantial risk of the spread of antibiotic resistance (14, 22), the usage of tylosin at subtherapeutic levels as a feed additive for growth promotion in food-producing animals was eventually banned in the EU in Council Regulation (EC) No. 2821/98 of 17 December 1998.

Today, the antibiotic is available under a broad range of commercial names, and has found clinical applications in the treatment of various bacterial conditions, including mycoplasmal diseases in major food-producing animals (13, 35). Tylosin is also among the antibiotics most widely used to treat infections caused by the Gram-negative anaerobes *Lawsonia intracellularis* (23) and *Brachyspira hyodysenteriae* (11), respectively the causative agents of porcine proliferative enteropathy and swine dysentery. However, research performed on field isolates from swine of the latter bacterium have demonstrated dwindling efficacy of tylosin (24, 36). Besides the antibiotic having found therapeutic applications in less obvious food-producing animals, it is used to control *Paenibacillus larvae*, the bacterium causing American foulbrood in the honey bee (31).

Regardless of the establishment of multiple analytical protocols allowing an affordable, accurate and rapid determination of tylosin (both exclusively and in a multianalyte method) in a wide variety of biological matrices including meat and offal (19, 27), dairy products (27), eggs (27), aquatic products (4), honey (19), feed (30), manure (12) and porcine oral fluid (9, 28), research evaluating the antibiotic concentration in sow’s milk cannot be found in scientific literature. Hence, the purpose of this study was to fill in the knowledge gaps and determine the concentration–time profile of tylosin in this matrix.

## Material and Methods

### Animal experiment

The research was conducted in 2024 on a commercial sow farm located in Poland. All the animals at the location were reared on a slatted floor under conditions meeting the requirements for the protection of pigs in Council Directive 2008/120/EC. The levels of protein, fibre and fat in the wheat- and barley-based pelleted lactation feed offered to the sampled sows were 15.2%, 4.4% and 4.7%, respectively. A group of five 1–3-year-old lactating sows weighing 190–260 kg and presenting the symptoms of arthritis was used to determine the penetration of tylosin into milk. All the animals involved in the trial were given intramuscular injections of tylosin by a veterinarian at 10 mg per kg of body weight for three consecutive days beginning on the first day postpartum. The tylosin used was Biotyl 200, in which the active substance is at a concentration of 200 mg/mL (Biowet Drwalew, Drwalew, Poland), and it was given at the dosage in the manufacturer’s recommendations. The withdrawal time for swine tissues declared by the drug manufacturer in the specification sheet was 21 d. Ethical review and approval were not required for this study, as the analysed material originated from a routine veterinary healthcare procedure ordered by the farm owners.

### Sample collection

Milk samples were collected manually by a veterinarian according to the following schedule: 3 h after, and then at fixed intervals of 1 d (24 h), 2 d (48 h), 5 d (120 h), 7 d (168 h), 14 d (336 h) and 21 d (504 h) after the first drug administration. Each 40 mL sample was collected into a sterile plastic 100 mL specimen jar, allowed to cool down, and stored at –19°C until the laboratory analysis. Prior to the treatment, a negative milk sample was collected from every animal involved in the study.

### Chemicals and standards

Tylosin tartrate was obtained from Dr. Ehrenstorfer (Augsburg, Germany). The internal standard (IS) of azithromycin and heptafluorobutyric acid (HFBA) were from Sigma-Aldrich (St. Louis, MO, USA). The acetonitrile used for sample extraction and mobile phase preparation was purchased from J.T. Baker (Deventer, the Netherlands). Hydrophilic polyvinylidene fluoride (PVDF, 0.22 μm) membrane syringe filters were purchased from Restek (Bellefonte, PA, USA). All reagents were analytically pure. Standard stock solutions of tylosin and azithromycin were prepared in methanol at a concentration of 1,000 μg/mL and stored for no longer than six months at ≤–18°C. Working solutions were prepared in ultrapure water and kept for no longer than one month at 4–8°C.

### Sample preparation

An aliquot of milk sample (2 mL) was weighed into a centrifuge tube, 100 μL of IS at the concentration of 2 μg/mL was added, and the mixture was vortexed and kept for 30 min at room temperature. For the extraction of tylosin, 8 mL of acetonitrile was used. Next, the samples were vortexed again for 15 min and centrifuged at 3,060 × g for 15 min at 4°C. Then, 6 mL of the upper layer of supernatant was transferred to a glass tube. The supernatant was evaporated to dryness under a stream of nitrogen at 45 ± 5°C. After evaporation, the sample was redissolved in 0.6 mL of 0.025% HFBA. Next, the sample was passed through 0.22 μm PVDF filters, transferred to an HPLC vial with a glass insert and analysed using ultra-high-performance liquid chromatography–tandem mass spectrometry (UHPLC-MS/MS).

### UHPLC-MS/MS conditions

Ultra-high performance liquid chromatography–tandem mass spectrometry analysis of tylosin in milk was carried out using a Shimadzu Nexera X2 UHPLC system (Shimadzu, Kyoto, Japan) coupled to a QTRAP 4500 triple quadrupole tandem mass spectrometer (AB Sciex, Framingham, MA, USA) with Analyst 1.6.2 software (AB Sciex). For chromatographic analysis, a Poroshell 120 end-capped (EC) C18 column, 150 mm × 2.1 mm × 2.7 μm (Agilent Technologies, Santa Clara, CA, USA) was used at a temperature of 35°C. The mobile phases were 0.025% HFBA in water (solution A) and acetonitrile (solution B), with the following concentration gradient: 0–5.0 min 5% mobile phase B, 5.01–8.00 min increasing to 80% of solvent B, and from 8.01 to 10.0 min decreasing to 50% of B. The oven temperature was set at 35°C with flow rate to 0.3 mL/min. The injection volume was 5 μL, and the run time was 10 min. The MS/MS analysis with the QTRAP 4500 triple quadrupole mass spectrometer was operated in the positive electrospray ionisation scan type with the multiple reaction monitoring mode (MRM), and the following parameter settings: nebuliser gas pressure of 60 psi; curtain gas pressure of 20 psi; ion spray voltage of 4,500 and temperature of 450°C. The ion transitions which were monitored were from 916 to 174/772 for tylosin and from 749 to 591 for azithromycin. The tylosin-specific mass spectrometric conditions were as follows: entrance potential (EP) 10 V, cell exit potential (CXP) 20 V (ion 1) and 14 V (ion 2), declustering potential (DP) 110 V and collision energy (CE) 52 V (ion 1) and 42 V (ion 2). For azithromycin as the IS, the following values were set: EP 10 V, CXP 13 V, DP 89 V and CE 40 V.

### Method validation

All validation parameters such as linearity, specificity, precision (repeatability and within-laboratory reproducibility) and trueness by recovery were analysed according to the criteria of Commission Regulation (EU) 2021/808 of 22 March 2021. Additionally, the limit of quantification (LOQ) was established as the lowest validated concentration with signal-to-noise ratio (S/N) of 10. The linearity was assessed based on a matrix calibration curve in three replicates by running concentration levels ranging from 5 to 3,000 μg/L. Specificity was evaluated by analysing 10 different blank milk samples to test the potential interference at the retention time of the analyte. The chromatograms of blank samples were compared to those of spiked samples. The coefficients of variation (CVs, %) at each fortification level were calculated in terms of repeatability and within-laboratory reproducibility, these being assessed at three different concentration levels – 5, 50 and 100 μg/L – by analysis of spiked samples in six replicates. The same operator was tasked with the analysis on the same day for repeatability, while for within-laboratory reproducibility another two sets of samples (n = 6) spiked with tylosin at the same three levels were analysed by different operators and on different days. The recovery was calculated in the same experiment as the repeatability.

## Results

### Validation of analytical methods

**Fig. 1.**
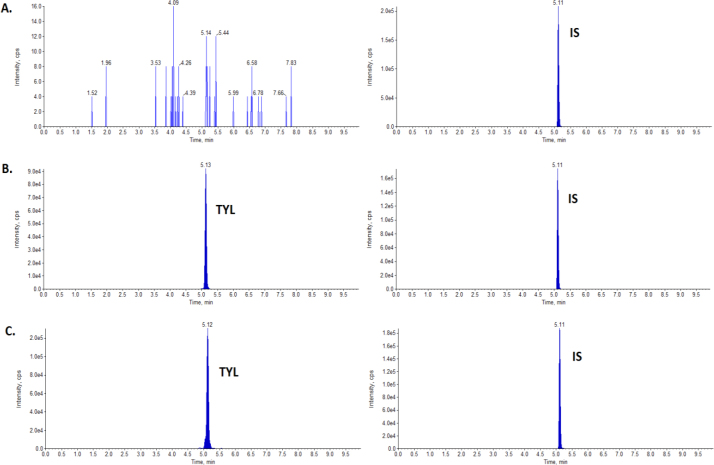
Ion chromatograms of A – a blank sow’s milk sample; B – a milk sample fortified with tylosin (TYL) at 50 μg/L; C – a milk sample at the concentration of 883 μg/L obtained one day after tylosin injection. cps – counts per s; IS – internal standard

The performed method for the quantitative analysis of tylosin in sow’s milk was sensitive, with satisfactory recovery and precision. The analyte of interest had excellent linearity for matrix-matched calibration curves, with r^2^ > 0.999. The results of specificity showed that no interference peak was observed in the chromatogram of blank milk samples compared to spiked samples at the retention time of the target analyte. The chromatograms of a blank sow’s milk sample, a sample fortified with tylosin at 50 μg/L and a sample collected one day after the end of treatment are presented in Fig. 1.

Precision and recovery were calculated using the IS and results inside the validation parameters were obtained. For the repeatability the coefficients of variation were lower than 10%, and for within-laboratory reproducibility they were lower than 15%. The recoveries ranged from 98.2 to 105.8, depending on the validation level. The achieved LOQ of 5 μg/L showed that the optimised method was sufficiently sensitive for measuring low levels of analyte in milk. The obtained validation results are listed in Table 1.

**Table 1.** Validation parameters of the method for the analysis of tylosin in sow’s milk

Analyte	Fortification level (μg/L)	Repeatability (%)	Reproducibility (%)	Recovery (%)
Tylosin	5.0	9.2	13.8	98.2
	50.0	7.0	6.7	105.8
	100.0	4.7	4.1	100.6

### Detection and Quantification of tylosin

**Fig. 2.**
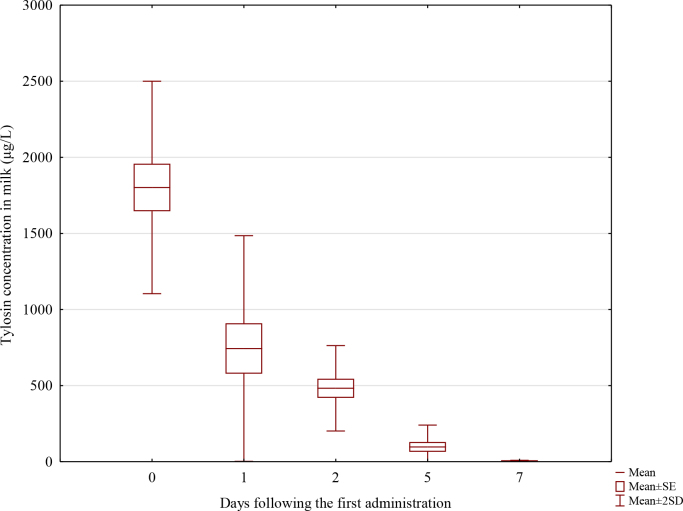
The tylosin concentration–time profile in sow’s milk (n = 5) following treatment consisting of three injectable daily doses of 10 mg of the antibiotic per kg of body weight. SE – standard error; SD – standard deviation

The concentration evaluated at seven time points confirmed the presence of tylosin in all the milk samples collected between 3 h and 7 d (168 h) after its first administration. The highest mean concentration of the substance, which was 1,802 μg/L (standard deviation (SD) 349), was found in the earliest samples. The mean levels of tylosin in the samples collected on day 1 (24 h) and d 2 (48 h) were 744 μg/L (SD 371) and 482 μg/L (SD 140), respectively. The mean concentration of the active substance in the material taken on day 5 (120 h) was markedly lower, falling to 97 μg/L (SD 72). The value of 6 μg/L (SD 2) in the samples collected on day 7 (168 h) constituted the lowest mean concentration of the analyte noted during the investigation. All the samples obtained 14 d (336 h) and 21 d (504 h) after the first administration contained the antibiotic in amounts below the LOQ. The tylosin concentration–time profile derived in the study is illustrated in Fig. 2.

## Discussion

Pharmacokinetics data on the transfer of tylosin into milk are restricted to several peer-reviewed works carried out using cows and less obvious dairy animals as study models (1, 8). It is relevant that apart from differing in the ruminant species included, the research frameworks in the experimental studies consisted of different administration routes, antibiotic dosages and sampling intervals. Moreover, as a natural consequence of practical focus on the treatment of the udder tissue infections in ruminants, the choice of animals involved in clinical trials was most often confined to those affected by mastitis. The lack of tabular data on actual residue concentrations of the antibiotic observed during a study and the adoption of different analytical procedures (including less accurate methods such as microbiological assays) in any such studies also severely limit direct comparisons. Even though the accumulated evidence seems to be radically influenced by all the aforementioned aspects, the data obtained in our study concurred well with previous findings demonstrating extensive penetration of tylosin to milk in ruminants.

As a weak lipophilic organic base, tylosin easily passes the blood–milk barrier, reaching its maximum concentration in a relatively short time. Indeed, as seen from our investigation, the highest mean concentration of the antibiotic among all the samples (1,802 μg/L; SD 349) was attained at 3 h following the administration of the therapy (Table 2). In a study involving clinically healthy goats which had received a single intramuscular dose of 10 mg of tylosin per kg of live weight, the antibiotic was detectable from 2 to 10 h after administration, at levels sharply declining along the timeline from 2,940 μg/L to 160 μg/L (1). Moreover, the concentration reported at 4 h (1,730 μg/L) closely matched the value obtained in our study at 3 h (1,802 μg/L). In another piece of research conducted on clinically healthy Slovak merino ewes injected with the same dose of the antibiotic for five consecutive days and milked for samples every 12 h, the highest mean concentration of tylosin (1,822 μg/L) was determined 12 h after the second administration (26).

**Table 2.** Tylosin residues in milk samples (μg/L) after the intramuscular administration of the antibiotic

Sampling time (h)	Goats (n = 5) (1)	Goats (n = 24) (32)	Ewes (n = 7) (26)	Sows (n = 5) (this study)
0	-*	-*	0*	0*
2	2,940	-	-	
3	-	-	-	1,802
4	1,730	-	-	-
6	700	-	-	-
8	390	-	-	-
10	160	-	-	-
12	0	-	632	-
24	-	-*	128*	371*
36	-	-	1,822	-
48	-	-*	471*	482*
60	-	-	1647	-
72	-	198	263*	-
84	-	-	1,046	-
96	-	NS	161*	-
108	-	-	900	-
120	-	NS	142	97
132	-	-	31	-
144	-	NS	0	-
156	-	-	-	-
168	-	0	-	6

1 - –no sample analysed; NS –positive but not specified;

* –intramuscular administration of 10 mg of tylosin per kg of live weight

In the present study, a rapid distribution of the antibiotic to sow’s milk was followed by its marked decline in this matrix to the levels of 744 μg/L (SD 371) on day 1 (24 h) and 482 μg/L (SD 140) on day 2 (48 h). Tylosin was also demonstrated to still be at measurable residual levels in samples collected on day 5 (120 h) and day 7 (168 h), but no residue of the antibiotic was found in the milk of any sow on day 14 (336 h) or day 21 (504 h). In other words, the last sampling time point when the residues were detected was 120 h after the last treatment. In previously cited research, the last sampling intervals at which tylosin could still be detected were 36 and 12 h after termination of the therapy in ewes and goats, respectively (1, 26). Nevertheless, research demonstrating a significantly longer decline time (96 h) in the latter species given a single dose of 10 mg of tylosin per kg is also on record (32).

It has been noted that in general, tylosin residues are depleted faster in small ruminants than they are in cows. Despite the dissimilarity in the doses of tylosin being given *via* intramuscular administration to heathy lactating cows in two pieces of research, a typical excretion time for the antibiotic evaluated in their milk could be defined and ranged from 24 to 96 h (2, 10). According to publicly available literature, local alterations of physicochemical parameters in the affected mammary gland (defined by a somatic cell count) were found to have influenced the pharmacokinetics of the antibiotic; cows which had contracted different types of mastitis exhibited significantly extended excretion periods, retaining measurable residual levels of tylosin in their milk up to 10 days (240 h) after termination of the therapy (8, 20).

As noted above, tylosin penetrated the udders of lactating sows well and relatively quickly when the pigs were given an intramuscular injection of the antibiotic. Modern scientific knowledge about the duration and magnitude of antibiotic milk levels in sows remains fairly rudimentary and is represented exclusively by some few peer-reviewed studies published in recent years (6, 7); therefore, our research contributes to the canon of knowledge of the comparative pharmacokinetics of the antibiotic. Nevertheless, a systematic analysis of the factors which may have influenced the distribution of the drug in lactating sows and its elimination from them merits further engagement.

Tylosin residues in different animal tissues have been extensively studied to date. Although the maximum residue limit (MRL) established by the EU authorities for muscle, fat, liver and kidney tissue (100 μg/kg; Commission Regulation No. 37/2010) is lower than the one set by the USA (200 μg/kg; Code of Federal Regulations Title 21 Part 556), the same criterion of 50 μg/kg for milk was adopted in both regions. As seen in our study, values exceeding the norm were observed from the first day of the investigation. Mean concentrations of the antibiotic in milk samples at or exceeding the MRL established for tylosin in cattle milk were noted between day 5 (120 h) and d 7 (168 h) after the commencement of the therapy. Given the human food safety assurance motivation for which the norms were set, extrapolation of the standards to suckling piglets ingesting contaminated sow’s milk is of debatable validity.

In the view of the ineffectiveness of tylosin against *Enterobacteriaceae* and the (erroneous) belief that sow’s milk concentrations of antibiotics in general were quantitatively negligible, the lack of previously published research on the discussed issue seems to be justified. It is noteworthy, however, that selected members of the macrolide group have been found to produce a variety of sub-inhibitory effects – meaning effects when used at weaker strengths than the minimum inhibitory concentration (MIC) – on selected bacteria, including one considerably hindering bacterial motility and adherence (33). Nevertheless, the available research recognised the biological effects of individual macrolides rather than the whole class, reported effects only with certain microorganisms, and gained them using highly specific experimental conditions and mostly in vitro models.

## Conclusion

The key findings and conclusions drawn in the literature cannot be extrapolated either to swine intestinal pathogens or to tylosin itself, because that research was not analogous to the present investigation. This being the case, the potential role of sub-MICs of tylosin in suckling piglets, including post-exposure effects on the development of intestinal microbiota, deserves further examination.
